# Single-cell RNA sequencing identifies two fibroblast subtypes and a Trem2^+^ macrophage subtype as the possible specific cellular targets in abdominal aortic aneurysms

**DOI:** 10.3389/fimmu.2025.1551308

**Published:** 2025-05-20

**Authors:** Zhili Liu, Xiaojun Song, Ben Wang, Rong Zeng, Liqiang Cui, Yuehong Zheng, Wei Ye

**Affiliations:** ^1^ Vascular Surgery Department, Chinese Academy of Medical Sciences, Peking Union Medical College Hospital, Beijing, China; ^2^ Department of Obstetrics and Gynecology, Daping Hospital, Third Military Medical University, Chongqing, China

**Keywords:** abdominal aortic aneurysm, crosstalk, fibroblasts, macrophage, single-cell RNA sequence

## Abstract

**Background:**

Aortic aneurysm is a potentially fatal condition. Although contemporary studies have established that this disease triggers an inflammatory response, reduces smooth muscle cells, and induces extracellular matrix remodeling, the involvement of inflammatory cells and associated signaling pathways in the progression and expansion of aneurysms is well-documented. However, clinical treatments utilizing anti-inflammatory therapies have proven ineffective.

**Methods:**

In this study, we employed a classic mouse model of abdominal aortic aneurysm (AAA) to compare the cellular composition and functional phenotypes of normal and AAA cells within a comprehensive single-cell microenvironment.

**Results:**

Our findings revealed distinct evolutionary pathways for both fibroblasts and macrophages, leading to the identification of specific fibroblast subtypes (Fib_Apoc1^+^/Fabp4^+^ and inflam-Fib1) and a macrophage subtype (Mac_TREM2). Cellular interactome analysis further reveals that fibroblasts and macrophages may play a certain synergistic role in the development of AAA. This study provides a comprehensive characterization of the transcriptional landscape of AAA and identifies novel therapeutic targets.

## Introduction

An abdominal aortic aneurysm (AAA) is characterized by a tumor-like dilation of the abdominal aorta, typically defined as an aneurysm with a diameter exceeding 50% of the normal size. The majority of cases are asymptomatic and challenging to detect in the early stages; however, ruptured aneurysms can be life-threatening. This represents a significant challenge in contemporary medicine, necessitating heightened awareness by both clinicians and researchers. The occurrence and development of AAA are often completely asymptomatic. In the majority of cases, AAA is diagnosed in patients presenting with aortic dissection or rupture. Therefore, it is essential to detect this disease promptly and carry out interventions. The epidemiological characteristics of AAA are closely related to cardiovascular diseases such as atherosclerosis, and it is more common in older men (1). The pathogenic factors of AAA are rather complex. Current research suggests that vascular inflammation, changes in the binding and content of vascular collagen, and the loss of vascular smooth muscle cells lead to changes in the aortic wall (2). Studying the characteristics of cytological changes in AAA and carrying out effective interventions are of great significance for public health and clinical practice (3).

Studies in human AAA tissues have shown that the density of medial smooth muscle cells (SMCs) is markedly reduced, accompanied by evidence of SMC apoptosis and elevated production of p53 (4). The degradation of elastin, infiltration of adaptive immune cells, and extracellular matrix proteolysis are hallmarks of AAA pathology (5). Inflammatory cells (6), such as macrophages (7), monocytes (8), neutrophils, NK cells, mast cells, and DCs, are essential determinants of aortic remodeling (9). Different subpopulations of macrophages have the same or opposite functions, such as “classically activated” M1 macrophages and “alternately activated” M2 macrophages. M1 macrophages are considered pro-inflammatory and M2 macrophages are considered anti-inflammatory, secreting many cytokines involved in extracellular stromal remodeling and tissue repair (10).

Although the contribution of inflammatory cells and associated signaling pathways to aneurysm development and expansion is well established, anti-inflammatory treatment methods have certain limitations and may need further contemplation. Fluoroquinolones, which are employed to treat a variety of infections, have even been associated with a heightened risk of aortic aneurysm and dissection (11). Studies of non-steroidal anti-inflammatory drugs (NSAIDs) have shown some effects. Celecoxib reduced aneurysm growth in Ang II-transfused ApoE^−/−^ mice; however, there have been no updated or new clinical trial results for nearly 20 years, which may be related to long-term side effects. Other anti-inflammatory treatments have also been shown to be ineffective, including anti-rheumatic drugs, steroids, and immunosuppressants, which are not effective in controlling the growth of AAA (9).

Given the poor effectiveness of anti-inflammatory therapy in abdominal aortic disease, we aim to conduct a comprehensive and detailed single-cell component analysis and cell differentiation study in the classic AAA mouse model. With the goal of providing patients with better surgical opportunities, we are trying to find new target cells and genes to control the continuous expansion of the abdominal aorta and reduce the possibility of AAA and the risk of rupture.

In this study, we conducted an unbiased high-throughput single-cell analysis on the ApoE^−/−^ angiotensin II-induced mouse model of AAA and analyzed cell-intrinsic signaling, cell–cell crosstalk, and cell-extrinsic factors at single-cell type resolution. We found that fibroblasts play a vital role through crosstalk, exerting an effect on macrophages. We identified two specific fibroblast subtypes, and the Fib_Apoc1^+^/Fabp4^+^ subtype was unique to AAA. The inflam-Fib1 subtype may be a crucial secretory subtype, recruiting inflammatory cells, and the Trem2^+^ macrophage subtype may be critical that occurs in an inflammatory response. The two fibroblast subtypes, Fib_Apoc1^+^/Fabp4^+^ and inflam-Fib1, and the Trem2^+^ macrophage subtype are specific target subtypes. We compare the transcriptomic profiles of normal and AAA tissues at single-cell resolution and reveal the changes in cell-intrinsic programs, cell–cell crosstalk, and cell-extrinsic factors during AAA formation, contributing to a deeper understanding of the critical role of fibroblasts in the formation of AAA.

## Materials and methods

### Data collection

We searched for “abdominal aortic aneurysm” in the Gene Expression Omnibus (GEO). We acquired three GEO single-cell RNAseq datasets (GSE239620, GSE221789, and GSE191226) and one bulk RNAseq dataset (GSE202267) after filtering.

### Quality control, data integration, and dimensionality reduction

The collected datasets were filtered based on the number of detected genes (between 300 and 8,000), the percentage of mitochondrial genes (less than 30%), the percentage of ribosome genes (more than 3%), and the percentage of red blood cell genes (less than 0.1%) to select qualified cells. Subsequently, we integrated multiple datasets covering control and AAA cells using the standard integration workflow as previously described (12).

### Differential gene expression analysis and functional annotation

The “FindAllMarkers” function in Seurat was utilized to detect differentially expressed genes (DEGs), with cutoff values set as an absolute fold change (|FC|) greater than 2 and an adjusted *p*-value less than 0.05. The GO enrichment analysis oof DEGs in this study was carried out using the clusterProfiler package. GSVA was conducted using the GSVA package (13). Differences between different cell groups were calculated by the “FindMarkers” function in the Seurat package. Gene ontology (GO) pathway enrichment analyses of DEGs were conducted by employing Metascape while keeping the default parameter settings. Subsequently, enrichment networks were generated and visualized automatically through the online tool Cytoscape. In this process, each enriched functional term was represented as a node, and pairs of nodes with a Kappa similarity score exceeding 0.3 were connected.

### Gene signatures

Inflammation-related genes such as *IFNG*, *IFNGR1*, *IFNGR2*, *IL10*, *IL12A*, *IL12B*, *IL12RB1*, *IL12RB2*, *IL13*, *IL17A*, *IL17F*, *IL18*, *IL18R1*, *IL18RAP*, *IL1A*, *IL1B*, *IL2*, *IL21*, *IL21R*, *IL22*, *IL23A*, *IL23R*, *IL2RG*, *IL4*, *IL4R*, *IL5*, *IL6*, *JUN*, *NFKB1*, *RELA*, *RORA*, *RORC*, *S100A8*, *S100A9*, *STAT1*, *STAT3*, *STAT4*, *STAT6*, *TGFB1*, *TGFB2*, *TGFB3*, and *TNF* were acquired from the previous publication authored by Smillie et al. (14).

### Cell–cell crosstalk analysis

CellChat inferred the potential intercellular communication, cell–cell contact, and cell–extracellular matrix interaction among diverse cell types based on the expression of known ligand–receptor pairs. This part of the method was carried out by referring to our previous analysis (12).

### Pathway analysis, pseudotime analysis by Monocle, and bulk RNA-seq analysis

This part of the method was implemented by referring to a previous publication by Zhang et al. (15).

### Immunohistochemistry staining

Formalin-fixed, paraffin-embedded normal and AAA specimens were obtained from Ang II-infused ApoE^−/−^ mice, as previously described (16). Immunohistochemistry (IHC) staining was performed as described previously (12).

### Statistical analysis

All statistical analyses were carried out using R (version 4.0.2). Statistical significance was assessed by the hypergeometric test or one-way ANOVA, which was then followed by the Bonferroni multiple comparison test. *p*-values and adjusted *p*-values that were lower than 0.05 were regarded as being statistically significant.

## Results

### Single-cell transcriptomic atlas and cell typing in normal and pathological abdominal aortas

**Figure 1:**
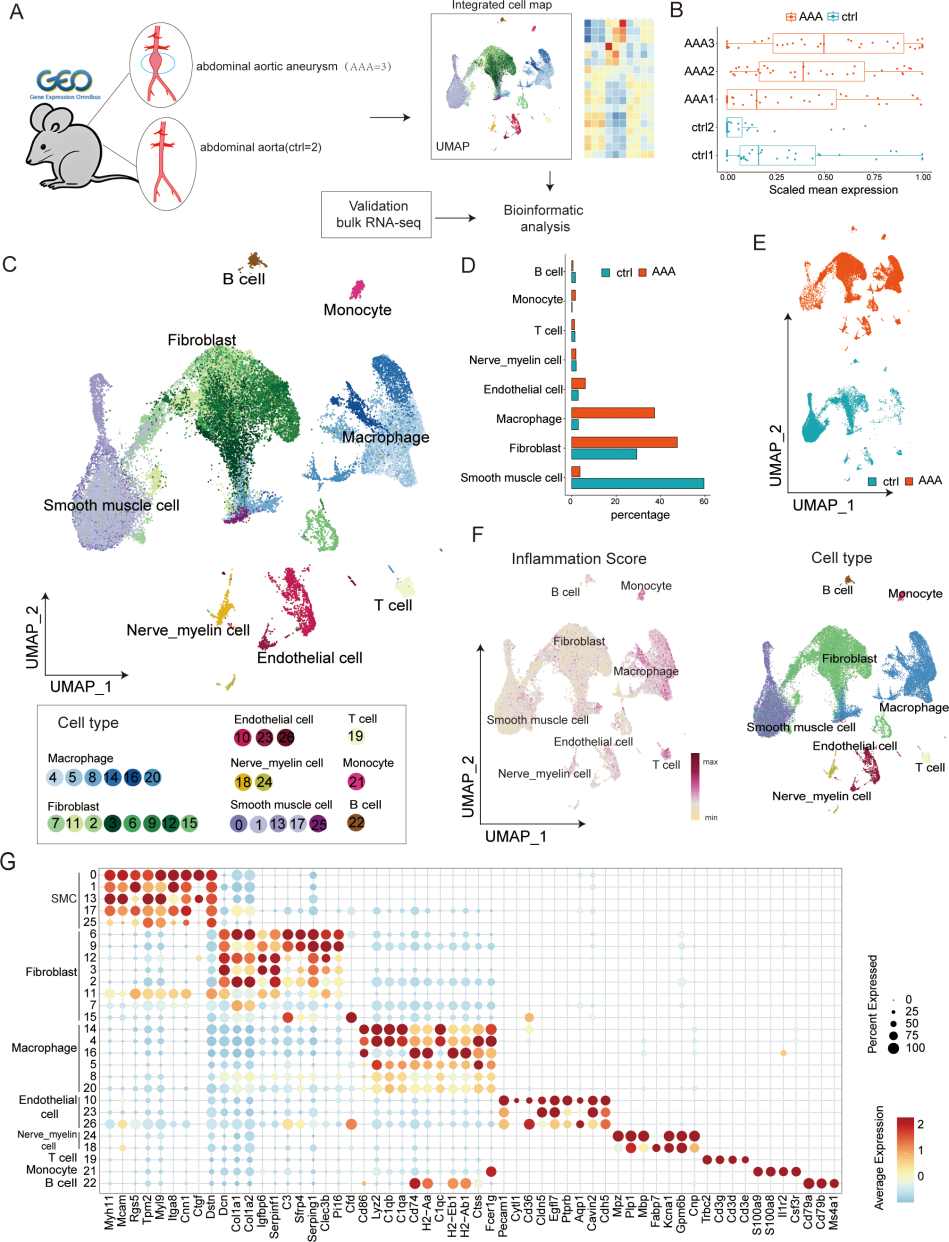
Single-cell transcriptome analysis in abdominal aortic aneurysm (AAA) and control samples. **(A)** Workflow of data analysis in this study. **(B)** Boxplots present the scaled mean expression of 42 inflammatory signatures in cells from different sample groups. In a boxplot, the internal horizontal line shows the median, the box covers the second to third quartiles, and the outer Tukey-style whiskers display the data dispersion. The dots denote individual signatures. **(C)** Uniform manifold approximation and projection (UMAP) plot presenting the integrated cell map, composed of 27 cell clusters from eight annotated cell types. Cells are colored by clusters. **(D)** Bar plot showing the cell-type abundance for samples from different groups. **(E)** UMAP visualization of 39,345 cells derived from AAA and control samples. **(F)** UMAP visualization of normal and AAA tissues colored by inflammation score (left) and cell types (right) in each cell type. **(G)** Dot plot showing representative marker genes across clusters. Dot size is proportional to the fraction of cells expressing specific genes. Color intensity corresponds to the relative expression level of specific genes.

It has been clearly demonstrated in AAA that inflammatory cells and related signaling pathways play a significant role in the development and expansion of aneurysms; however, anti-inflammatory treatment methods have exhibited certain limitations that call for further thought and exploration. We attempted a more detailed single-cell analysis using a classic mouse model of AAA. To re-analyze the pathogenesis of AAA from the perspective of differentiation of major cells, we collected single-cell transcriptome datasets from the three published Ang II-induced AAA mouse models, including five samples from the AAA group and the control group. **Figure 1A** illustrates the general experimental process. Following multiple quality control steps, we acquired single-cell transcriptomes in a total of 39,345 cells from all samples for downstream analysis. These cells were partitioned into 27 clusters of eight main cell types, namely, SMC (clusters 0, 1, 13, 17, 25; *Acta2*, *Actg2*, *Mcam*, *Myl9*, *Myh11*), fibroblasts (clusters 7, 11, 2, 3, 6, 9, 12, 15; *Dcn*, *Col1a1*, *Col1a2*), macrophages (clusters 4, 5, 8, 14, 16, 20; *C1qb*, *C1qa*, *Cd86*), endothelial cells (clusters 10, 23, 26; *Cldn5*, *Pecam1*, *Eng*), monocytes (cluster 21; *S100a8*, *S100a9*), T cells (cluster 19; *Cd3d*, *Cd3e*, *Cd3g*), B cells (cluster 22; *Cd79a*, *Cd79b*, *Ms4a1*), and nerve_myelin cells (clusters 24, 18) (**Figure 1C**, **Supplementary Figure S1A**). All cells were annotated with known markers (**Figure 1G**, **Supplementary Figure S1D**) that were consistent with other previous articles. Because *Cnp*, *Gpm6b*, *Fabp7*, *Mbp*, and *Mpz* are mainly expressed in nerve and myelin cells, we define clusters 24 and 18 as nerve_myelin cells.

Given the essential role of inflammatory reactions in AAA, we used the previously determined gene signatures to score the inflammatory gene expression for five samples and eight cell types in our dataset (**Figures 1B, F**, right). The result showed that AAA had higher inflammatory scores, and clusters of macrophages, monocytes, and T cells were most closely related to inflammation, which was consistent with previous studies. Then, we compared the changes in the relative abundance of different cells. We found that SMC, inflammatory cells, and fibroblasts changed the most (**Figures 1D, E**, **Supplementary Figure S1C**). Existing research has already indicated that this disease involves changes in the extracellular matrix, a decrease in smooth muscle cells, and an infiltration of inflammatory cells. However, there is little research on the role of fibroblasts in this disease. We speculate that fibroblasts may play a relatively important bridging role between inflammatory cells and smooth muscle cells.

### Macrophages may be the most essential inflammatory cells, and NF-κB signaling may represent a key inflammatory pathway in AAA

**Figure 2:**
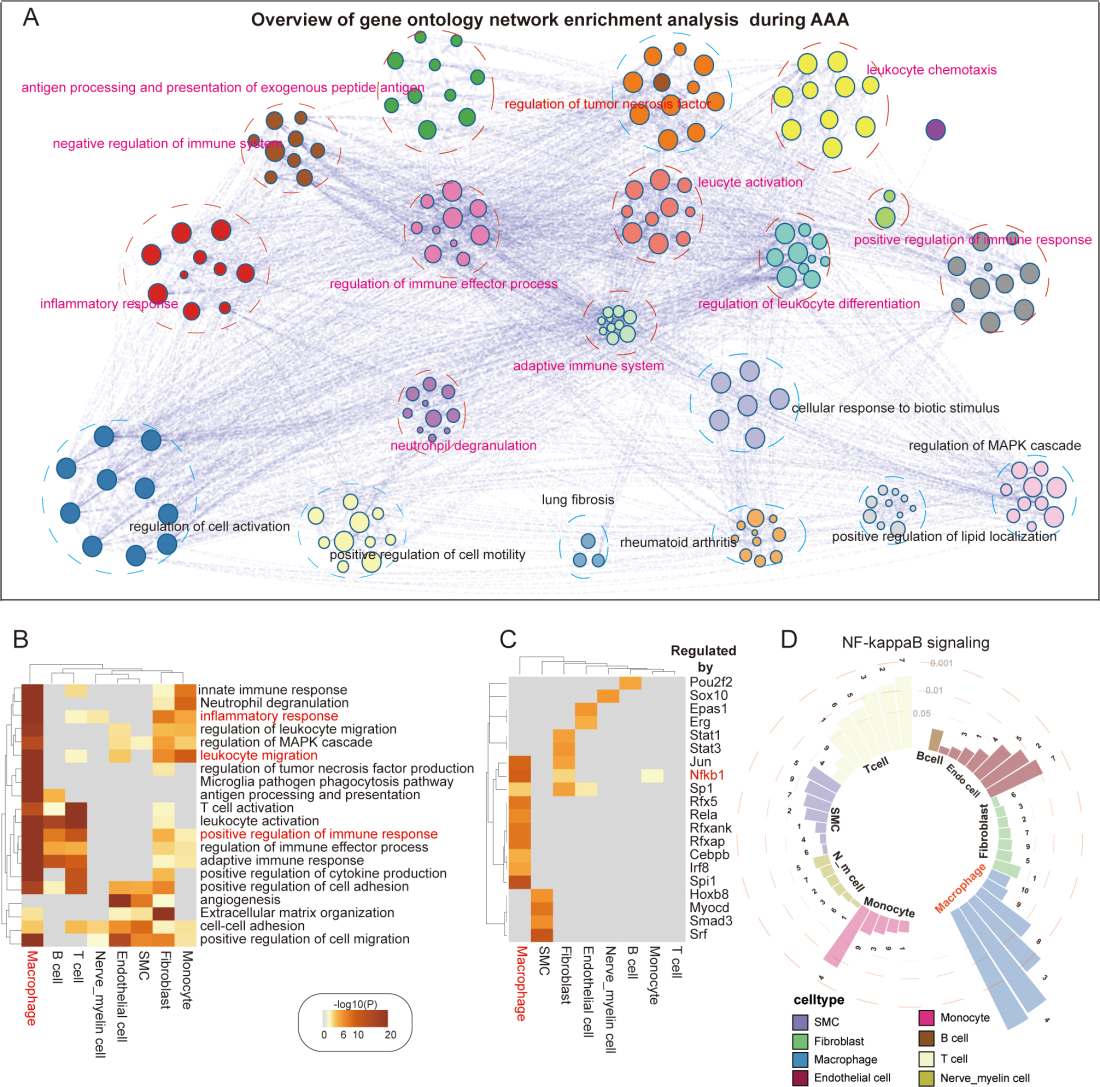
AAA-related transcriptomic changes in abdominal aorta. **(A)** Overview of gene ontology network enrichment analysis across eight cell clusters during AAA formation. Each node stands for a specific gene ontology term. Similar terms are grouped and combined for the purpose of annotation. The linkage between nodes indicates the protein–protein interaction. The gene ontology interaction network was obtained by functional enrichment analysis using Metascape. **(B)** Heatmap showing the top 20 common biological processes enriched in AAA cells. **(C)** Heatmap showing the top 20 hub genes regulating specific genetic programs during AAA formation. **(D)** Ring bar plot showing the activation of the NF-κB signaling pathway in diverse abdominal aortic cell types. Circular dashed lines represent the value of adjusted *p*-values, which are 0.05, 0.01, and 0.001, respectively. Numbers 1–10 represent different NF-κB signaling pathways (1. I-κB kinase/NF-κB signaling; 2. NIK/NF-κB signaling; 3. positive regulation of I-κB kinase/NF-κB signaling; 4. positive regulation of NF-κB transcription factor activity; 5. positive regulation of NIK/NF-κB signaling; 6. regulation of I-κB kinase/NF-κB signaling; 7. regulation of NIK/NF-κB signaling; 8. negative regulation of I-κB kinase/NF-κB signaling; 9. negative regulation of NF-κB transcription factor activity; 10. negative regulation of NIK/NF-κB signaling).

Many studies have verified the essential role of inflammatory reactions in AAA, including macrophages, monocytes, NK cells, B cells, and T cells. We wanted to know which immune cells play a very critical role in AAA. To achieve this goal, we systematically investigated AAA-related shifts in biological processes, performed cell type-specific differential gene expression analysis between control and AAA groups, and identified AAA-related differentially expressed genes. Afterward, we developed a comprehensive enrichment network in which each node represents a single biological process (**Figure 2A**, **Supplementary Table S4**). Gene ontology analysis of DEGs verified and broadened the shared biological processes that exist among different cell types, such as the inflammatory response, leukocyte migration, adaptive response, and extracellular matrix organization (**Figure 2B**). In our analysis, we found that macrophages took part in all immune-related pathways during AAA formation, including cytokine production, innate immune response, and adaptive immune response. Fibroblasts are most closely related to the formation of the extracellular matrix (**Figure 2B**).

Then, we wanted to know which pathway is the most important inflammatory pathway. By using an alternative method based on TRRUST, we analyzed the transcriptional regulatory interactions of DEGs with Metascape and found 20 transcriptional regulators, suggesting that SP1, CEBPB, and JUN act as shared or cell type-specific hub genes that may regulate AAA-dependent transcriptional changes (**Figure 2C**). Some studies have verified that the NF-κB pathway is very important for AAA formation (17). Our analysis of upstream transcription factors of AAA also showed that the activity of NF-κB1 of macrophages was the highest. We surprisingly found that fibroblasts also had high transcriptional activity (**Figure 2C**). Considering the potential role that NF-κB plays in the formation of systemic AAA, we investigated 10 signaling pathways involving NF-κB and discovered that these pathways were generally activated in the majority of cell types, especially in macrophages (**Figure 2D**).

### Cell–cell crosstalk patterns reshaped by AAA formation

**Figure 3:**
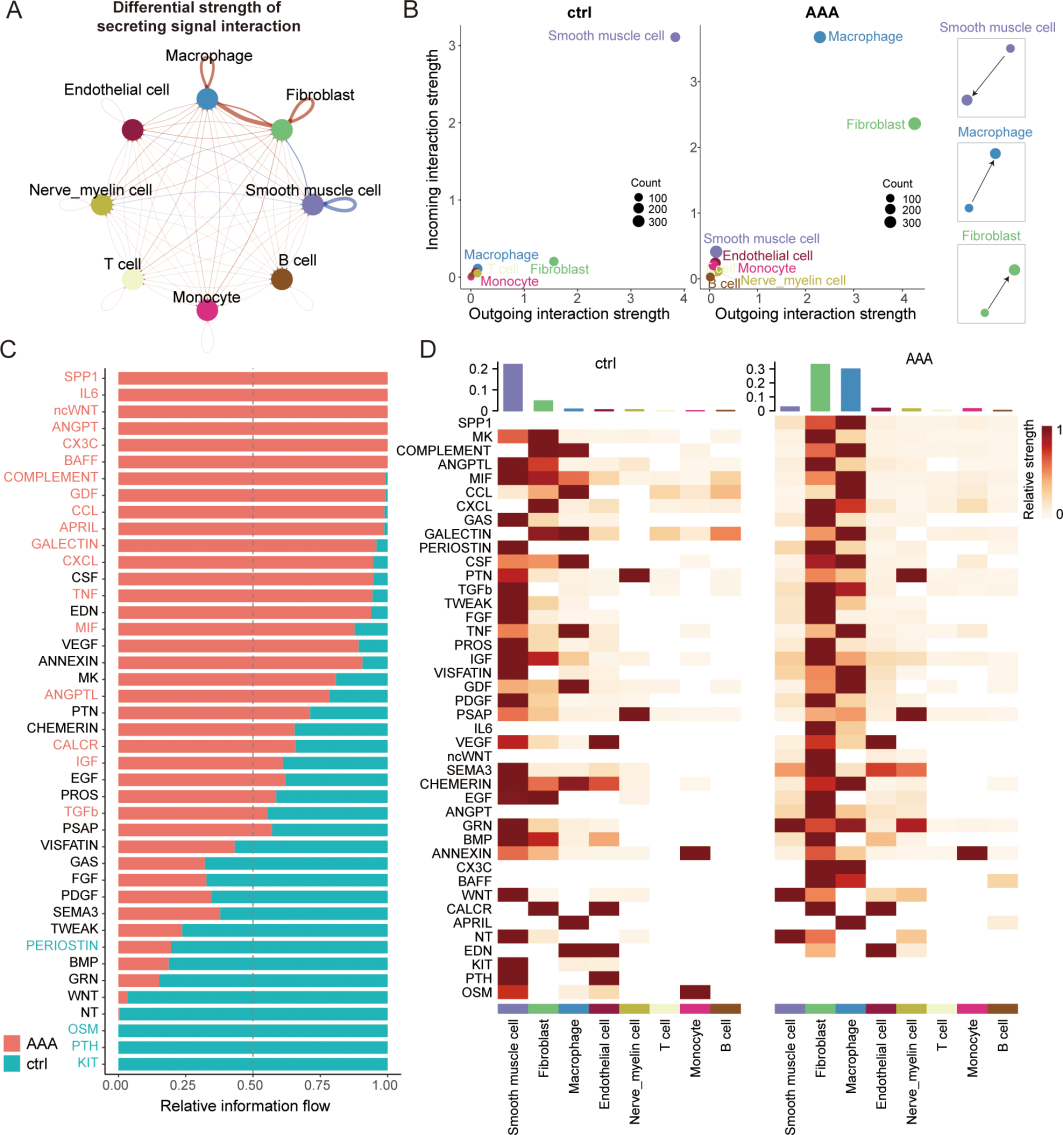
Altered cell–cell crosstalk during AAA formation. **(A)** Circle plot showing the differential strength of secreting signal interaction among eight different cell types in AAA and control abdominal aortic tissue. The strength of cell–cell communication correlates with the transcriptional expression of significant ligand–receptor pairs, and the width of the edges connecting the two cell types is proportional to the strength of the ligand–receptor pairs. Red and blue lines represent enriched cell–cell interactions in AAA and normal cells, respectively. **(B)** Scatter plot showing the relative outgoing and incoming interaction strength of secreted signaling for each of the cell types in the control (ctrl) and AAA cells. Scaled-down schemes on the right highlight the increased outgoing signal interaction in fibroblasts/macrophages and the reduced incoming signal interaction on SMC during AAA formation. **(C)** Ranked bar plot showing the secretory factor-mediated signaling pathways enriched in either normal or AAA cells. The relative strength of information flow for each signaling pathway is defined by the sum of the communication probabilities among all pairs of cell groups. The secretory factors highlighted in red were enriched in AAA cells. **(D)** Heatmap shows the relative strength of secretory factor-mediated signaling pathways in diverse cell types within AAA and control (ctrl) samples. SMCs, smooth muscle cells.

Existing studies have shown that the clinical effect of anti-inflammatory therapy is limited, so we wanted to evaluate the specific cells in the development of AAA from the perspective of intercellular communication and investigate whether other cell types may also be key targets for AAA treatment. We first generated a comprehensive map of cell–cell communications to depict the difference between normal and AAA cells in secreting signal interactions using CellChat (**Figure 3A**).

While the role of macrophages in AAA has long been established, we have also found something different by conducting comprehensive CellChat analyses. Overall, we found that fibroblasts may be the most powerful secretory signaling cells in AAA, and macrophages are the strongest signal-receiving cells (**Figures 3B–D**, **Supplementary Figure S3B**). Current studies of fibroblasts in AAA have mainly focused on the extracellular matrix (18) and myofibroblast vascular remodeling (19), but our results show that fibroblasts can have a specific relationship with macrophages through crosstalk. This is a relatively interesting finding, and we wanted to see if fibroblasts have some of the same characteristics in inflammatory pathways. Therefore, we then compared some of the major inflammatory pathways of AAA and found that the TNF and TGFb signaling pathways are mainly sent by macrophages and received by fibroblasts, while the CCL, ANGPTL, COMPLEMENT, and CXCL signaling pathways are mainly sent by fibroblasts and received by macrophages (**Supplementary Figure S3C**). All these findings suggest that fibroblasts may play a very specific role in AAA immune infiltration through crosstalk.

Existing studies in AAA have found that myofibroblasts can produce chemokines and cytokines, but there is no single-cell transcriptome dataset for AAA to analyze fibroblasts in more detail. Through CellChat analysis, we speculated that fibroblasts may be the specific secretory cells in the occurrence of AAA. These fibroblasts may regulate the progression of AAA. Meanwhile, fibroblasts have the function of migration, and there are many subgroups. Therefore, we wanted to focus on analyzing fibroblast subsets and macrophage subsets to explore possible drug target cells.

### Two fibroblast subtypes show their crucial roles in AAA

**Figure 4:**
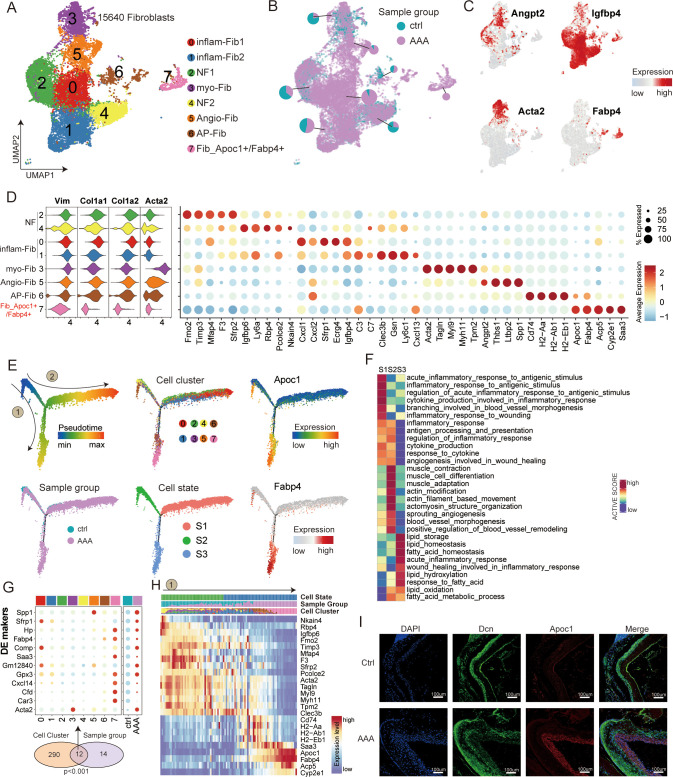
Transcriptional profiling of fibroblasts in AAA and control samples. **(A)** UMAP showing the subtypes of fibroblasts, including inflam-Fib1, inflam-Fib2, NF1, myo-Fib, NF2, Angio-Fib, AP-Fib, and Fib_Apoc1^+^/Fabp4^+^, colored by subclusters. **(B)** Distribution of fibroblasts in different sample groups on the UMAP. Pie chart showing the proportion of two sample groups in each subcluster. **(C)** Feature plots showing the expression of selected cluster-specific genes. Cells with the highest expression level are colored red. **(D)** Violin plots (left) display the representative expression pattern across different subtypes of fibroblasts. Dot plotsw (right) showing the expression of the top subtype-specific gene markers in each subtype. **(E)** Semi-supervised pseudotime trajectory of fibroblast subtypes by Monocle2. The trajectory is colored by pseudotime (top left), sample groups (bottom left), cell clusters (top middle), cell states (bottom middle), and expression dynamics of the two marker genes Apoc1 and Fabp4 (right). **(F)** Heatmap showing the functional pathways enriched in three cell states (S1–S3) of fibroblasts by GSVA analysis. **(G)** Venn diagram (bottom) showing the overlap of DEGs between subclusters and sample groups of fibroblasts. The *p*-value was calculated using the *χ*² test. Dot plot (top) showing the expression of these 12 DEGs across all cell subclusters and sample groups. **(H)** Heatmap illustrating the gene dynamics along the pseudotime of route 1. Bar plots above the heatmap are scaled diagrams of different cell states, sample groups, and cell clusters during pseudotime differentiation trajectory. **(I)** Immunofluorescence staining showing the co-localization of Dcn (green), Apoc1 (red), and DAPI (blue) in control and AAA samples. Scale bars, 100 μm.

**Figure 5:**
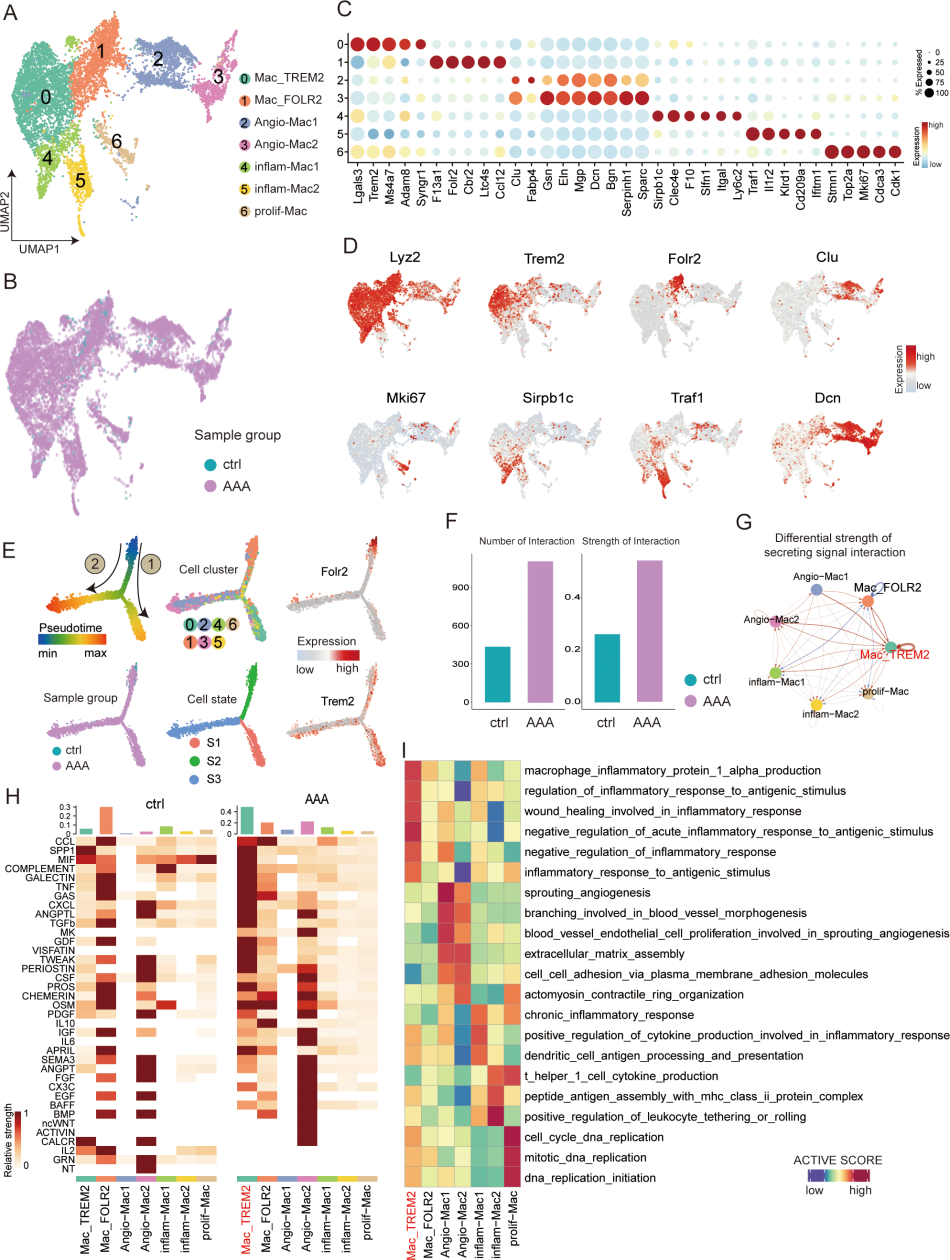
Transcriptional profiling of macrophages in AAA and control samples. **(A)** UMAP showing the subtypes of macrophages, including Mac_TREM2, Mac_FOLR2, neg-Mac, Angio-Mac, inflam-Mac1, inflam-Mac2, and prolif-Mac, colored by subclusters. **(B)** Distribution of macrophages in different sample groups on the UMAP. **(C)** Dot plot showing the expression of the top subtype-specific gene markers in each subtype. **(D)** Feature plots showing the expression of selected cluster-specific genes. Cells with the highest expression level are colored red. **(E)** Semisupervised pseudotime trajectory of macrophage subtypes by Monocle2. The trajectory is colored by pseudotime (top left), sample groups (bottom left), cell clusters (top middle), cell states (bottom middle), and expression dynamics of the two marker genes Folr2 and Trem2 (right). **(F)** Bar plot showing the overall view of all macrophages of different groups: the comparison of the quantity and intensity of communication. **(G)** Circle plot showing the differential strength of secreting signal interaction among seven different subtypes of macrophages in AAA and control samples. **(H)** Heatmap shows the relative strength of secretory factor-mediated signaling pathways among seven different subtypes of macrophages in AAA and control (ctrl) samples. **(I)** Heatmap showing the functional pathways enriched in seven different subtypes of macrophages by GSVA analysis.

First, we focused on the analysis of fibroblasts (**Figure 4A**). It was found that all of the fibroblast clusters exhibited a high level of expression of the well-established pan-fibroblast markers, such as *COL1A1*, *COL1A2*, *DCN*, and *VIM* (**Figures 4A, B**). Fibroblasts are known to constitute the majority of the desmoplastic stroma by regulating extracellular matrix components in AAA. We identified eight subtypes in our data based on known marker genes (**Figures 4C, D**). Cluster 0 was defined as inflam-Fib1 based on the expression of classical inflammatory markers such as *Cxcl1*, *Cxcl2*, *Sfrp1*, and *Igfbp*4 (as shown by immunofluorescence staining, **Figure 5F**); cluster 1 was defined as inflam-Fib2 based on the expression of inflammatory markers such as *C3*, *C7*, and *Cxcl13*; cluster 3 was defined as myCAFs based on the expression of classical myofibroblastic markers such as *Acta2* (*αSMA*), *Myl9*, and *Myh11*; clusters 2 and 4 were for normal fibroblasts (NF1 and NF2); cluster 5 was defined as an angiogenic fibroblast (Angio-Fib; *Angpt2* and *Thbs1*); and cluster 6 was defined as an antigen-presenting fibroblast (AP-Fib; *H2-Ab1* and *H2-Eb1*). Of the eight fibroblast clusters, cluster 7 (Fib_Apoc1^+^/Fabp4^+^) contained the unique subtype that existed only in the AAA group. Furthermore, the co-expression of *Dcn/Apoc1* and *Dcn/Fabp4* was validated by multicolor immunofluorescence staining in both normal and AAA mouse tissues, confirming the existence of Fib_Apoc1^+^/Fabp4^+^ in AAA (**Figure 4I**, **Supplementary Figure S4E**).

To investigate the developmental mechanisms and potential roles played by these different fibroblast subpopulations in AAA, we carried out a cell trajectory analysis to analyze their progression route. We rearranged the fibroblasts into pseudotime trajectories by utilizing Monocle2 and defined the normal cluster as the starting point. This revealed three distinct cell states (S1–S3) and two major trajectory routes (routes 1 and 2; **Figures 4E, G, H**, **Supplementary Figure S4B**).

Interestingly, we noted that the outcome of these two kinds of trajectory routes is quite different, among which the endpoint of route 1 is Fib_Apoc1^+^/Fabp4^+^, and the differentiation state is from S2 to S3, which is mainly related to lipid metabolism (including lipid storage and lipid oxidation). Many researchers have long found that hypertension, hypercholesterolemia, and obesity are significantly associated with AAA. This may also be indirectly explained by our discovery of route 1, which is associated with a significantly enhanced lipid metabolism pathway. Route 2 is mainly directed toward inflammatory-associated cells, including clusters 0 and 1 (inflam-Fib1, inflam-Fib2). The differentiation state of cells along route 2 goes from S2 to S1. We hypothesized that inflammatory fibroblasts of pathway 2 may secrete some useful inflammatory factors to recruit inflammatory cells to infiltrate AAA. In summary, our analysis revealed the differentiation characteristics of fibroblast subtypes, and both terminally differentiated cell types may be potential therapeutic target cells.

### The Trem2 macrophage subtype may play a specific role in AAA

**Figure 6:**
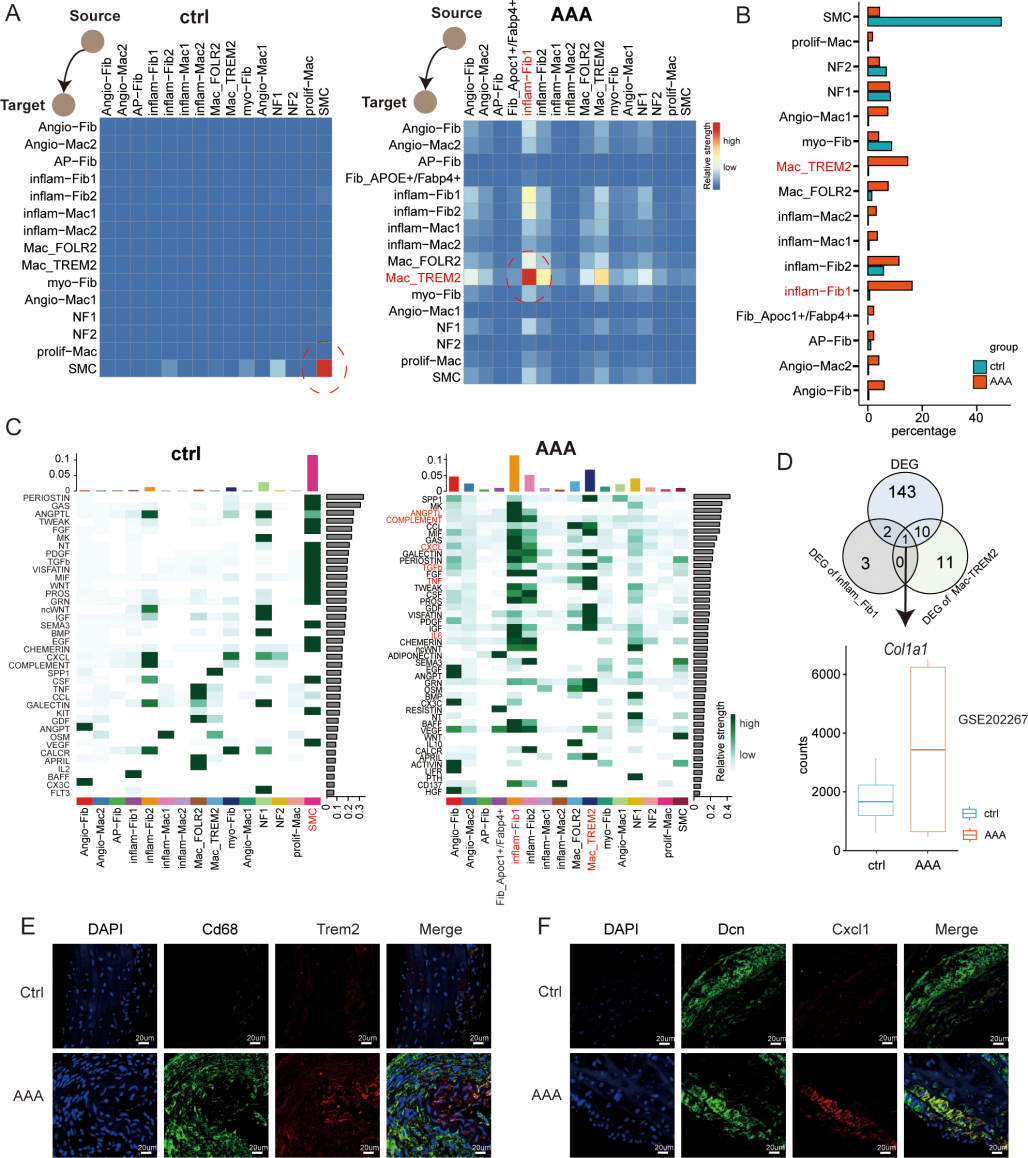
Dynamics of cell–cell interaction networks during AAA formation. **(A)** Heatmap illustrating the cell–cell interaction patterns in control (ctrl) and AAA samples. **(B)** Bar plot showing the percentage of cells in each subtype across macrophages, fibroblasts, and SMCs. **(C)** Heatmap shows the relative strength of secretory factor-mediated signaling pathways of subtypes within AAA and control (ctrl) samples. The expression levels are represented by the intensity of colors. SMCs, smooth muscle cells. **(D)** Venn diagrams showing the overlap of the up-DEGs in total DEGs, inflam-Fib1, and Mac_TREM2 cells. Boxplots showing the expression patterns of Col1a1 using the bulk RNA-seq dataset of GSE202267. **(E)** Immunofluorescence staining showing the co-localization of CD68 (green), Trem2 (red), and DAPI (blue) in control and AAA samples. Scale bars, 20 µm. **(F)** mmunofluorescence staining showing the co-localization of Dcn (green), Cxcl1 (red), and DAPI (blue) in control and AAA samples. Scale bars, 20 µm.

Because macrophages may have the greatest inflammatory impact in AAA disease and may play a crucial role in the progression of the disease, we wanted to conduct a more detailed analysis of the subtypes of macrophages to understand the differentiation characteristics of AAA during the occurrence. We partitioned the 8,962 macrophages obtained from different mouse sources into seven distinct cell subsets (**Figures 5A, B, D**, **Supplementary Figure S5A**). It is relatively simple to divide macrophages into M1 and M2 using single cells. So, we identified seven subtypes based on marker genes and enrichment pathways (**Figures 5C, I**). Cluster 0 was defined as Mac_TREM2 based on the expression of the classical macrophage marker Trem2 (as shown by immunofluorescence staining, **Figure 6E**); cluster 1 was defined as Mac_FOLR2 based on the high expression of *Folr2*; clusters 2 and 3 were defined as Angio-Mac based on the enrichment pathway (sprouting angiogenesis and blood vessel morphogenesis); clusters 4 and 5 were for inflammatory subtypes (inflam-Mac1 and inflam-Mac2; *Igtal*, *Slfn1*, and *Il2r*); and cluster 6 was defined as a proliferative subtype (prolif-Mac; *Mki67* and *Cdk1*). Moreover, the co-expression of *Cd68/Trem2* was validated by multicolor immunofluorescence staining in both normal and AAA mouse tissues, confirming the existence of Mac_TREM2 in AAA (**Figure 6E**).

To describe the evolutionary dynamics of macrophage lineages during the progression of AAA, we conducted unsupervised cell trajectory analysis with the help of Monocle2. The pseudotime trajectory analysis based on Monocle2 identified three cell states (S1–S3) and two major trajectory routes (routes 1 and 2; see **Figure 5E**, **Supplementary Figure S5C**). Route 1 mainly lead to the development of the Mac_TREM2 subtype, while route 2 mainly lead to the development of vascular macrophages.

We wanted to know which subtype of macrophage plays the specific role, so we generated a comprehensive map of cell–cell communication to depict the difference of macrophages in secreting signal interactions using CellChat (**Figures 5F–H**). As we can see, the Mac_TREM2 subtype is the most active, and the vast majority of inflammatory pathways are concentrated in Mac_TREM2. At the same time, Angio-Mac2 can secrete a variety of angiogenic factors, such as endothelial growth factor (EGF) and fibroblast growth factor (FGF), and these factors can stimulate the proliferation, migration, and lumen formation of endothelial cells, thus promoting the formation of new blood vessels (**Figure 5H**). Angiogenesis is a crucial part of vascular damage itself. Regulation of angiogenesis by vascular macrophages to meet the tissue demand for oxygen and nutrients is the direction of route 2. We hypothesized that route 1 is a pathway that aggravates inflammation and injury, while route 2 plays a protective role such as M2 macrophages through angiogenesis and other route.

### Landscape of the reprogramming interactome of fibroblasts, macrophages, and smooth muscle cells in AAA

Finally, to understand the crosstalk between normal tissues and those affected by AAA within the microenvironment during the progression of AAA, we made use of a public database of ligand–receptor (L-R) interactions from CellChat to visualize the intercellular interactions. We wanted to know during the occurrence of AAA, in the reprogramming process among these three types of cells, which cell is the specific one and how they influence each other. Through cell communication, we can see from **Figure 6A** that when AAA occurs, inflam-Fib1 releases signals, while Mac-TREM2 receives signals. Current research has already demonstrated that macrophages may play a very important role in AAA. We have presented the two specific cell subtypes. It can also be seen from **Figure 6B** that when the disease occurs, the difference between these two groups of cells is the greatest. Subsequently, we compared the changes in the pathways among different cell types, and we can see that the CCL/COMPLEMENT/TGFb/TNF pathway signalings were mainly observed in the cells of the AAA group. The Venn diagram showed the differentially expressed genes of AAA, imflam-Fib1, and Mac-TREM2 (**Figure 6D**). The gene we finally identified was *Col1a1*, which significantly interferedwith cell crosstalk. Together, these data provide mechanistic insights, from the perspective of intercellular interactions, into the formation of the main environment of AAA.

## Discussion

Although the important role of inflammatory factors in AAA has been confirmed by many studies, the clinical effect of anti-inflammatory therapy is very limited (9). Fibroblasts have been confirmed to have cell proliferation during AAA formation (20). However, current studies mostly regulate the progression of AAA from the perspective of myofibroblasts (19). To date, very few studies have focused on exploring the relevance of fibroblasts to inflammatory cells (21) or the relevance of fibroblasts to SMC using single-cell sequencing methods (22). Given that we have confirmed the importance of inflammatory factors in this disease, fibroblasts may also be a specific target associated with inflammation that should not be overlooked. In this study, our objective was to present a comprehensive single-cell transcriptomic overview for depicting the microenvironment in AAA by utilizing classic AAA mouse models, with the aim of thoroughly investigating the mechanisms underlying the occurrence of AAA. Our study not only provides a high-resolution portrayal of the cellular diversity among fibroblasts, macrophages, and SMC components, but also highlights the intercellular crosstalk in both normal conditions and in the context of AAA. Finally, we found two fibroblast subtypes (Fib_Apoc1^+^/Fabp4^+^ and inflam-Fib1) and the Trem2^+^ macrophage subtype, that may be effective cellular targets for the treatment of AAA.

We used cellular interactome analysis to reveal for the first time that fibroblasts and macrophages may play a synergistic role in the development of AAA, which is consistent with the results of the recent experimental study by Markus et al. (23). Nevertheless, compared with immune cells and smooth muscle cells, fibroblasts have received relatively less research attention. Fibroblasts can facilitate the formation of the extracellular stroma and maintain its structural integrity. Research has shown that adventitial fibroblasts can transform into activated myofibroblasts, which are not typically found in healthy blood vessels (24). Fibroblasts transform into myofibroblasts through the expression of alpha-smooth muscle actin (α-SMA) (25). Myofibroblasts are mobile, and their overactivation or persistence can lead to pathological vascular remodeling (19). In our study, cluster 3 was myCAFs thatfollowed route 2 (**Figure 4E**). Through single-cell sequencing, we found an inflammatory fibroblast subtype (inflam-Fib1) with relatively high expression of *Cxcl1* (**Figure 6F**), and through crosstalk (**Figure 4E**), it was verified that this subtype was most closely related to macrophages. Surprisingly, we identified a subpopulation of fibroblasts (Apoc1^+^/Fabp4^+^) predominantly expressed in AAA tissues, which was validated by immunofluorescence staining. Fib_Apoc1^+^/Fabp4^+^ was first found in our study. Importantly, pseudotime trajectory analysis revealed Fib_Apoc1^+^/Fabp4^+^ to be in the direction of lipid metabolism (route 1, **Figure 4E**). Hypertension, hypercholesterolemia, and obesity (26) have all been found to be significantly associated with AAA (27). Therefore, the Fib_Apoc1^+^/Fabp4^+^ subpopulation may be a potential therapeutic target for AAA patients. The precise role of Fib_Apoc1^+^/Fabp4^+^ in AAA progression needs to be further explored.

Macrophages represent the most abundant immune cells in the aortic wall and play a crucial role in aortic wall inflammation (28). The single-cell map of macrophages in aortic aneurysm has been reported (16), which also reported Trem2 macrophage subpopulations. Another study reported an *Il1rn* ^+^/*Trem1* ^+^ macrophage subpopulation in thoracic aortic aneurysm and dissection, and *Il1rn* and *Trem1* were also relatively highly expressed in the Mac_TREM2 subtype we found (16). Through unbiased clustering of single-cell transcriptomes, we identified seven macrophage subpopulations in the AAA tissues. We noted that the Mac_TREM2 subtype was the strongest inflammatory group, and Mac_FOLR2 was dominant in normal tissue. Through cell trajectory analysis, route 2 shows Angio-Mac2 to be associated with angiogenesis, which may produce a variety of angiogenic factors, such as EGF and FGF; however, notably, several studies have shown that angiogenesis is a characteristic change that may promote AAA occurrence, progression, and rupture (29). Therefore, whether Angio-Mac2 is a therapeutic target needs to be further explored.

There are some limitations to our study. First, we only selected the downstream data of the classic angiotensin II-induced mouse model in GEO, but the quality of some downstream data we screened was not good, and the sample size was only five cases in the end. Therefore, our data may not reflect the entire ecosystem of AAA, nor can it fully simulate the pathogenesis characteristics of AAA in another type of mouse model or humans. Furthermore, the analysis of ligand–receptor interactions between different cell components is mainly based on transcriptome prediction. These predicted interactions need to be further validated by high-dimensional multiplex *in situ* analysis.

In summary, our study offers a detailed and profound characterization of the transcriptomic as well as functional phenotypes of the various cellular components within the microenvironment of AAA mouse samples. The crosstalk among fibroblasts, macrophages, and SMCs is also emphasized, which helps to promote a deeper comprehension of the underlying mechanisms. Moreover, further functional verification of our analyses is necessary, and our analysis can serve as a valuable resource for the design of targeted therapies for AAA.

## Data Availability

The datasets analyzed for this study can be found in the Gene Expression Omnibus (GEO): three GEO Single-cell RNAseq datasets (GSE239620, GSE221789, and GSE191226) and one bulk RNAseq dataset (GSE202267).

## Supplementary material

Supplementary Figure 1 Comprehensive dissection and clustering of 39,345 single cells from abdominal aortic tissues. Related to **Figure 1**. **(A)** UMAP plot of 39,345 cells colored by the cell clusters. **(B)** UMAP plot of 39,345 cells colored by samples. **(C)** Bar plot showing the percentage of cells in each group across all major cell types. **(D)**Heatmap showing the relative expression level of specific marker genes (left). Examples of marker gene expression are shown in the right UMAPs

Supplementary Figure 2 Bubble chart to display the ligand-receptor-mediated cell interaction relationships among fibroblasts, smooth muscle cells, and macrophages. The dot color and size represent the calculated communication probability and p-values. SMC, smooth muscle cells.

Supplementary Figure 3
**(A)** Circle plot showing the differential strength of cell-cell contact interaction among 8 different cell types in control and AAA’s abdominal aorta. **(B)** Scatter plot showing the relative outgoing and incoming interaction strength of secreted signaling for each of the cell types in control (ctrl) and AAA’s (AAA) abdominal aorta. **(C)** Circle plots showing the enhancement of TGFb-/TNF-/ANGPTL-/CCL/COMPLEMENT/CXCL signaling network for each of cell types in control (ctrl) and AAA’s (AAA) abdominal aorta. Each colored dot represents individual cell types, and the width of edges connecting circles represents the strength of intercellular signaling.

Supplementary Figure 4
**(A)** UMAP visualization of fibroblasts derived from normal and AAA’s abdominal aorta. **(B)** Heatmap illustrating the gene dynamics along the pseudotime of route 2. Bar plots above the heatmap are scaled diagrams of different cell states, sample groups, and cell clusters during pseudotime differentiation trajectory. **(C)** Bar plot showing subtypes of fibroblast abundance for samples from different groups, the routes are marked above. **(D)** Compare the semisupervised pseudotime trajectories of fibroblast subtypes according to different groups. **(E)** Immunofluorescent staining showing co-localization of Dcn (green), Fabp4 (red), and DAPI (blue) in control and AAA samples. Scale bars, 20 μm.

Supplementary Figure 5
**(A)** UMAP visualization of macrophages derived from normal and AAA’s abdominal aorta. **(B)** Bar plot showing subtypes of macrophage abundance for samples from different groups. **(C)** Heatmap illustrating the gene dynamics of macrophages along the pseudotime of route 2. Bar plots above the heatmap are scaled diagrams of different cell states, sample groups, and cell clusters during pseudotime differentiation trajectory. **(D)** Heatmap showing the functional pathways enriched in three cell states (S1–S3) of macrophages by GSVA analysis.

Supplementary Figure 6
**(A)**Circle plots showing the enhancement of CCL-/COMPLEMENT-/TGFb-/TNF- signaling network for each of subtypes in control (ctrl) and AAA’s (AAA) abdominal aorta. Each colored dot represents individual cell types, and the width of edges connecting circles represents the strength of intercellular signaling. **(B)** Heatmap showing the top 50 hub genes regulating specific genetic programs during AAA formation.

## References

[1] Quintana RA Taylor WR. Introduction to the compendium on aortic aneurysms. Circ Res. (2019) 124:470–1. doi: 10.1161/CIRCRESAHA.119.314765 30763220

[2] Jana S Hu M Shen M Kassiri Z. Extracellular matrix, regional heterogeneity of the aorta, and aortic aneurysm. Exp Mol Med. (2019) 51:1–15. doi: 10.1038/s12276-019-0286-3 PMC6923362 31857579

[3] Kapila V Jetty P Wooster D Vucemilo V Dubois L. Screening for abdominal aortic aneurysms in Canada: 2020 review and position statement of the Canadian Society for Vascular Surgery. Can J Surg. (2021) 64:E461–6. doi: 10.1503/cjs.009120 PMC8526155 34467750

[4] López-Candales A Holmes DR Liao S Scott MJ Wickline SA Thompson RW. Decreased vascular smooth muscle cell density in medial degeneration of human abdominal aortic aneurysms. Am J Pathol. (1997) 150:993–1007. 9060837 PMC1857880

[5] Shen YH LeMaire SA Webb NR Cassis LA Daugherty A. Lu HS. Part I: dynamics of aortic cells and extracellular matrix in aortic aneurysms and dissections. Arterioscler Thromb Vasc Biol. (2020) 40:e37–46. doi: 10.1161/ATVBAHA.120.313991 PMC7233726 32101472

[6] Jana S Chute M Hu M Winkelaar G Owen CA Oudit GY. ADAM (a disintegrin and metalloproteinase) 15 deficiency exacerbates ang II (Angiotensin II)–induced aortic remodeling leading to abdominal aortic aneurysm. Arterioscler Thromb Vasc Biol. (2020) 40:1918–34. doi: 10.1161/ATVBAHA.120.314600 PMC7370975 32522006

[7] Turner GH Olzinski AR Bernard RE Aravindhan K Boyle RJ Newman MJ. Assessment of macrophage infiltration in a Murine model of abdominal aortic aneurysm. J Magn Reson Imaging. (2009) 30:455–60. doi: 10.1002/jmri.21843 19629967

[8] Wu S Liu S Wang B Li M Cheng C Zhang H. Single-cell transcriptome in silico analysis reveals conserved regulatory programs in macrophages/monocytes of abdominal aortic aneurysm from multiple mouse models and human. Front Cardiovasc Med. (2023) 9:1062106. doi: 10.3389/fcvm.2022.1062106 36698942 PMC9868255

[9] Pi S Xiong S Yuan Y Deng H. The role of inflammasome in abdominal aortic aneurysm and its potential drugs. Int J Mol Sci. (2024) 25:5001. doi: 10.3390/ijms25095001 38732221 PMC11084561

[10] Willemsen L de Winther MP. Macrophage subsets in atherosclerosis as defined by single-cell technologies. J Pathol. (2020) 250:705–14. doi: 10.1002/path.5392 PMC7217201 32003464

[11] Zhang J Zhang Z. Fluoroquinolones increase the risk of aortic aneurysm and dissection: A protocol for meta-analysis. Med (Baltimore). (2021) 100:e28081. doi: 10.1097/MD.0000000000028081 PMC8702248 34941048

[12] Chen Z-H Li S Xu M Liu CC Ye H Wang B. Single-cell transcriptomic profiling of the hypothalamic median eminence during aging. J Genet Genomics. (2022) 49:523–36. doi: 10.1016/j.jgg.2022.01.001 35032691

[13] Hänzelmann S Castelo R Guinney J. GSVA: gene set variation analysis for microarray and RNA-seq data. BMC Bioinf. (2013) 14:7. doi: 10.1186/1471-2105-14-7 PMC3618321 23323831

[14] Smillie CS Biton M Ordovas-Montañes J Sullivan KM Burgin G Graham DB. Cellular and inter-cellular rewiring of the human colon during ulcerative colitis. Cell. (2019) 178:714–730.e22. doi: 10.1016/j.cell.2019.06.029 31348891 PMC6662628

[15] Zhang S Fang W Zhou S Zhu D Chen R Gao X. Single cell transcriptomic analyses implicate an immunosuppressive tumor microenvironment in pancreatic cancer liver metastasis. Nat Commun. (2023) 14:5123. doi: 10.1038/s41467-023-40727-7 37612267 PMC10447466

[16] Le S Wu J Liu H Du Y Wang D Luo J. Single-cell RNA sequencing identifies interferon-inducible monocytes/macrophages as a cellular target for mitigating the progression of abdominal aortic aneurysm and rupture risk. Cardiovasc Res. (2024) 120:1351–64. doi: 10.1093/cvr/cvae117 38836630

[17] Ren J Han Y Ren T Fang H Xu X Lun Y. AEBP1 promotes the occurrence and development of abdominal aortic aneurysm by modulating inflammation via the NF-κB pathway. J Atheroscler Thromb. (2020) 27:255–70. doi: 10.5551/jat.49106 PMC7113137 31462616

[18] Mackay CDA Jadli AS Fedak PWM Patel VB. Adventitial fibroblasts in aortic aneurysm: unraveling pathogenic contributions to vascular disease. Diagnostics. (2022) 12:871. doi: 10.3390/diagnostics12040871 35453919 PMC9025866

[19] Han X Wu A Wang J Chang H Zhao Y Zhang Y. Activation and migration of adventitial fibroblasts contributes to vascular remodeling. Anat Rec. (2018) 301:1216–23. doi: 10.1002/ar.23793 29406614

[20] Liu X Chen W Zhu G Yang H Li W Luo M. Single-cell RNA sequencing identifies an Il1rn+/Trem1+ macrophage subpopulation as a cellular target for mitigating the progression of thoracic aortic aneurysm and dissection. Cell Discov. (2022) 8:11. doi: 10.1038/s41421-021-00362-2 35132073 PMC8821555

[21] Xie C Hu Y Yin Z. Inhibiting YAP1 reduced abdominal aortic aneurysm formation by suppressing adventitial fibroblast phenotype transformation and migration. J Cell Mol Med. (2024) 28:e70159. doi: 10.1111/jcmm.70159 39495769 PMC11534076

[22] Xiong J Chen G Lin B Zhong L Jiang X Lu H. Integrative analysis of single-Cell RNA sequencing and experimental validation in the study of abdominal aortic aneurysm progression. Gene. (2024) 929:148820. doi: 10.1016/j.gene.2024.148820 39103059

[23] Wagenhäuser MU Mulorz J Krott KJ Bosbach A Feige T Rhee YH. Crosstalk of platelets with macrophages and fibroblasts aggravates inflammation, aortic wall stiffening, and osteopontin release in abdominal aortic aneurysm. Cardiovasc Res. (2024) 120:417–32. doi: 10.1093/cvr/cvad168 37976180

[24] Tinajero MG Gotlieb AI. Recent developments in vascular adventitial pathobiology: the dynamic adventitia as a complex regulator of vascular disease. Am J Pathol. (2020) 190:520–34. doi: 10.1016/j.ajpath.2019.10.021 31866347

[25] Coen M Gabbiani G Bochaton-Piallat M-L. Myofibroblast-mediated adventitial remodeling: an underestimated player in arterial pathology. Arterioscler Thromb Vasc Biol. (2011) 31:2391–6. doi: 10.1161/ATVBAHA.111.231548 21868702

[26] Lu F Lin Y Zhou J Chen Z Liu Y Zhong M. Obesity and the obesity paradox in abdominal aortic aneurysm. Front Endocrinol. (2024) 15:1410369. doi: 10.3389/fendo.2024.1410369 PMC11269098 39055063

[27] Song P He Y Adeloye D Zhu Y Ye X Yi Q. Global health epidemiology research group (GHERG). The global and regional prevalence of abdominal aortic aneurysms: A systematic review and modeling analysis. Ann Surg. (2023) 277:912–9. doi: 10.1097/SLA.0000000000005716 PMC10174099 36177847

[28] Wang X Zhang H Cao L He Y Ma A Guo W. The role of macrophages in aortic dissection. Front Physiol. (2020) 11:54. doi: 10.3389/fphys.2020.00054 32116765 PMC7013038

[29] Jia Y Li D Yu J Jiang W Liu Y Li F. Angiogenesis in aortic aneurysm and dissection: A literature review. Rev Cardiovasc Med. (2023) 24:223. doi: 10.31083/j.rcm2408223 39076698 PMC11266809

