# Supplementary figures and images for: Single-cell RNA sequencing identifies two fibroblast subtypes and a Trem2+ macrophage subtype as the possible specific cellular targets in abdominal aortic aneurysms
Source: Front Immunol. 2025 May 20;16:1551308. doi: 10.3389/fimmu.2025.1551308 (PMC12131870; doi:10.3389/fimmu.2025.1551308)

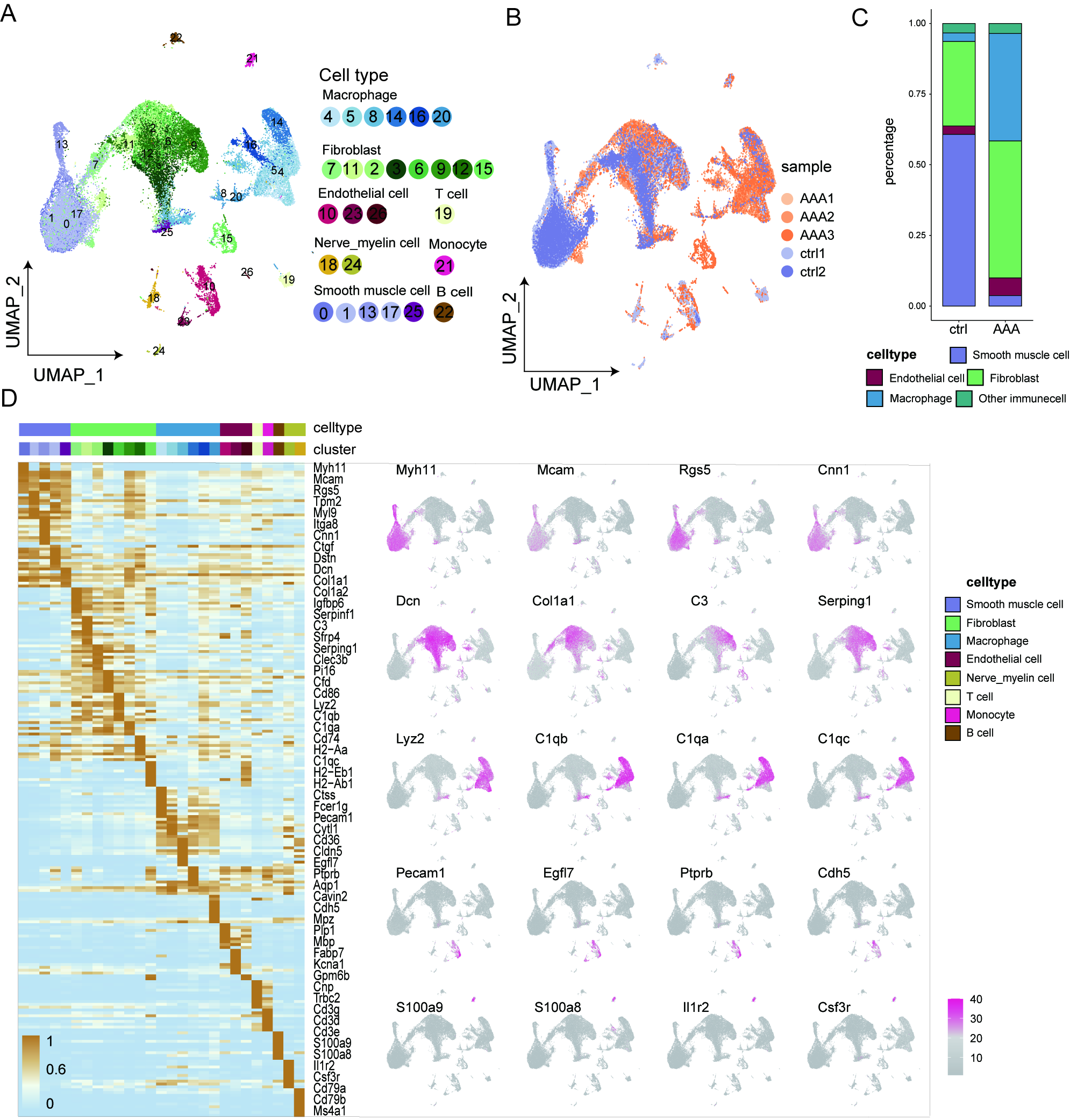

Supplement: Supplementary Figure 1 — Comprehensive dissection and clustering of 39,345 single cells from abdominal aortic tissues. Related to Figure 1 . (A) UMAP plot of 39,345 cells colored by the cell clusters. (B) UMAP plot of 39,345 cells colored by samples. (C) Bar plot showing the percentage of cells in each group across all major cell types. (D)Heatmap showing the relative expression level of specific marker genes (left). Examples of marker gene expression are shown in the right UMAPs [file Image1.tif]

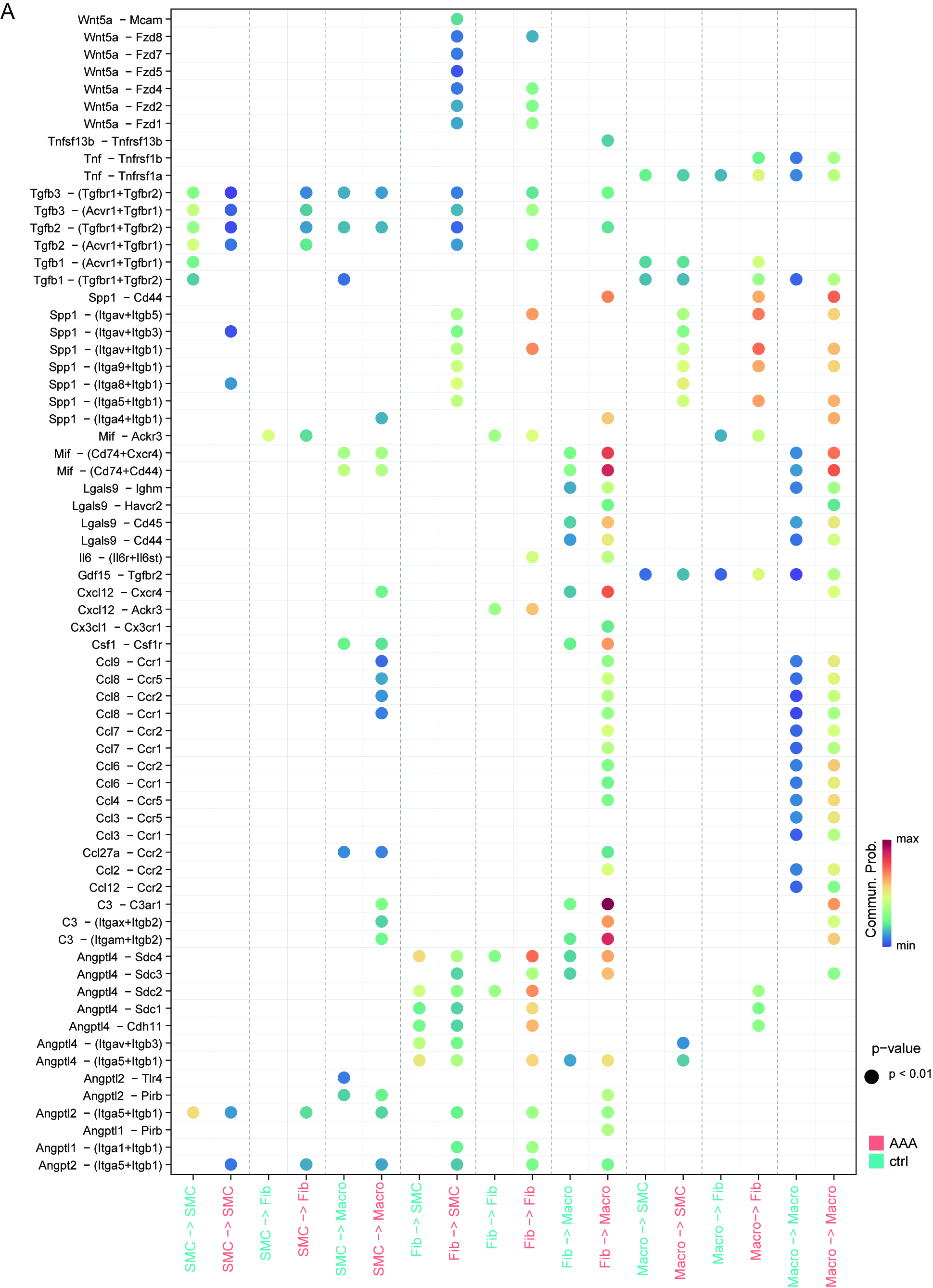

Supplement: Supplementary Figure 2 — Bubble chart to display the ligand-receptor-mediated cell interaction relationships among fibroblasts, smooth muscle cells, and macrophages. The dot color and size represent the calculated communication probability and p-values. SMC, smooth muscle cells. [file Image2.tif]

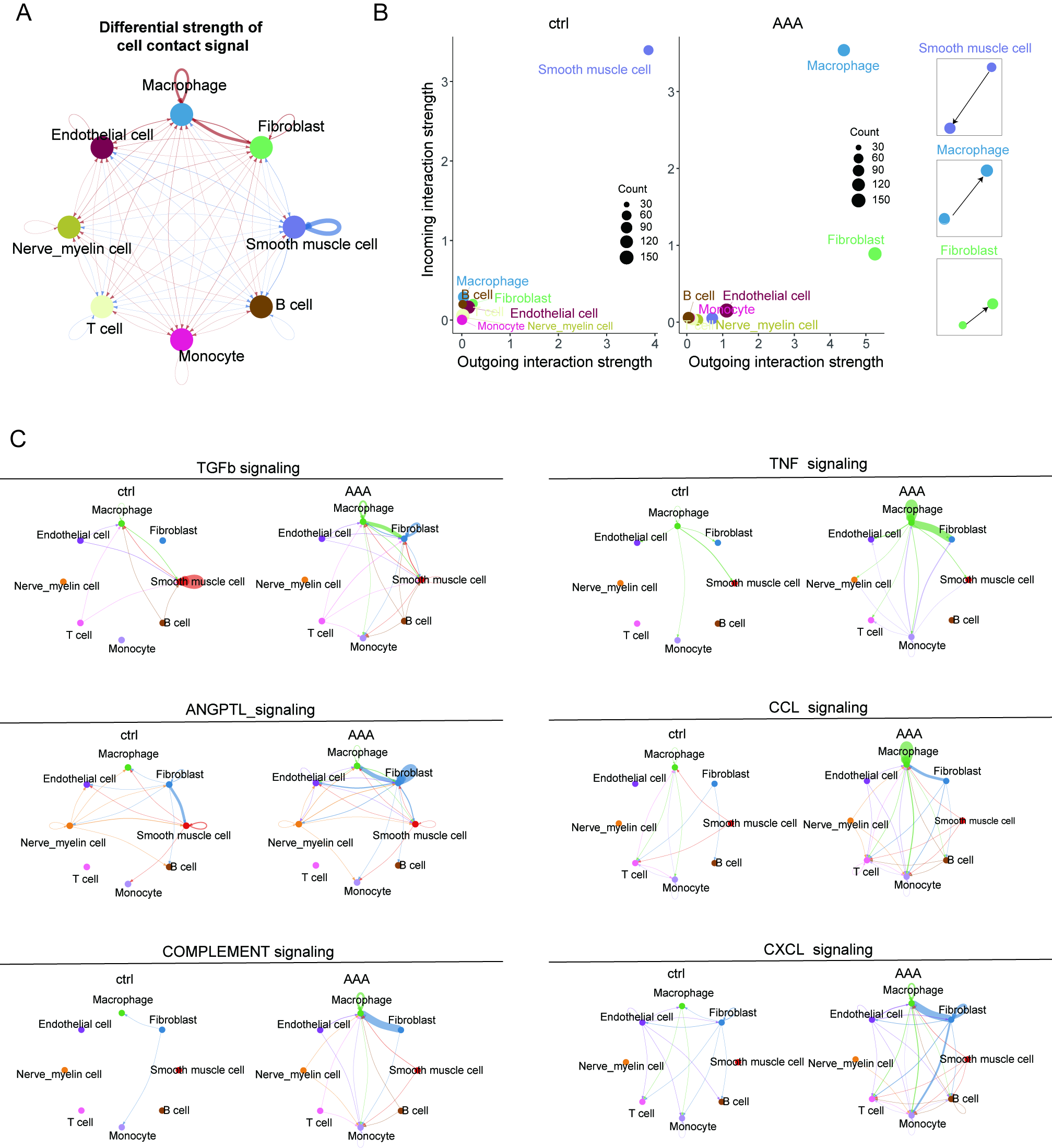

Supplement: Supplementary Figure 3 — (A) Circle plot showing the differential strength of cell-cell contact interaction among 8 different cell types in control and AAA’s abdominal aorta. (B) Scatter plot showing the relative outgoing and incoming interaction strength of secreted signaling for each of the cell types in control (ctrl) and AAA’s (AAA) abdominal aorta. (C) Circle plots showing the enhancement of TGFb-/TNF-/ANGPTL-/CCL/COMPLEMENT/CXCL signaling network for each of cell types in control (ctrl) and AAA’s (AAA) abdominal aorta. Each colored dot represents individual cell types, and the width of edges connecting circles represents the strength of intercellular signaling. [file Image3.tif]

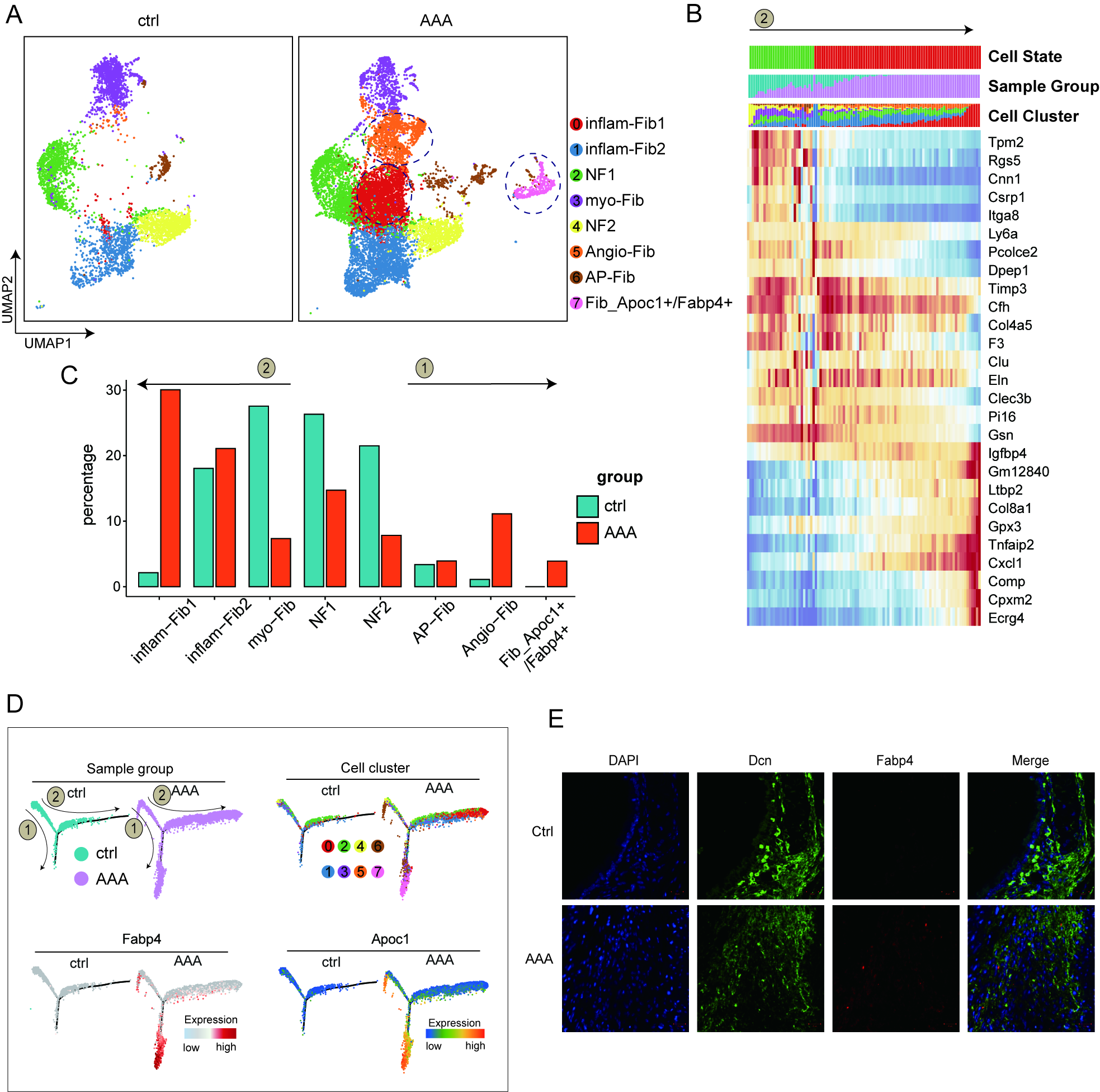

Supplement: Supplementary Figure 4 — (A) UMAP visualization of fibroblasts derived from normal and AAA’s abdominal aorta. (B) Heatmap illustrating the gene dynamics along the pseudotime of route 2. Bar plots above the heatmap are scaled diagrams of different cell states, sample groups, and cell clusters during pseudotime differentiation trajectory. (C) Bar plot showing subtypes of fibroblast abundance for samples from different groups, the routes are marked above. (D) Compare the semisupervised pseudotime trajectories of fibroblast subtypes according to different groups. (E) Immunofluorescent staining showing co-localization of Dcn (green), Fabp4 (red), and DAPI (blue) in control and AAA samples. Scale bars, 20 μm. [file Image4.tif]

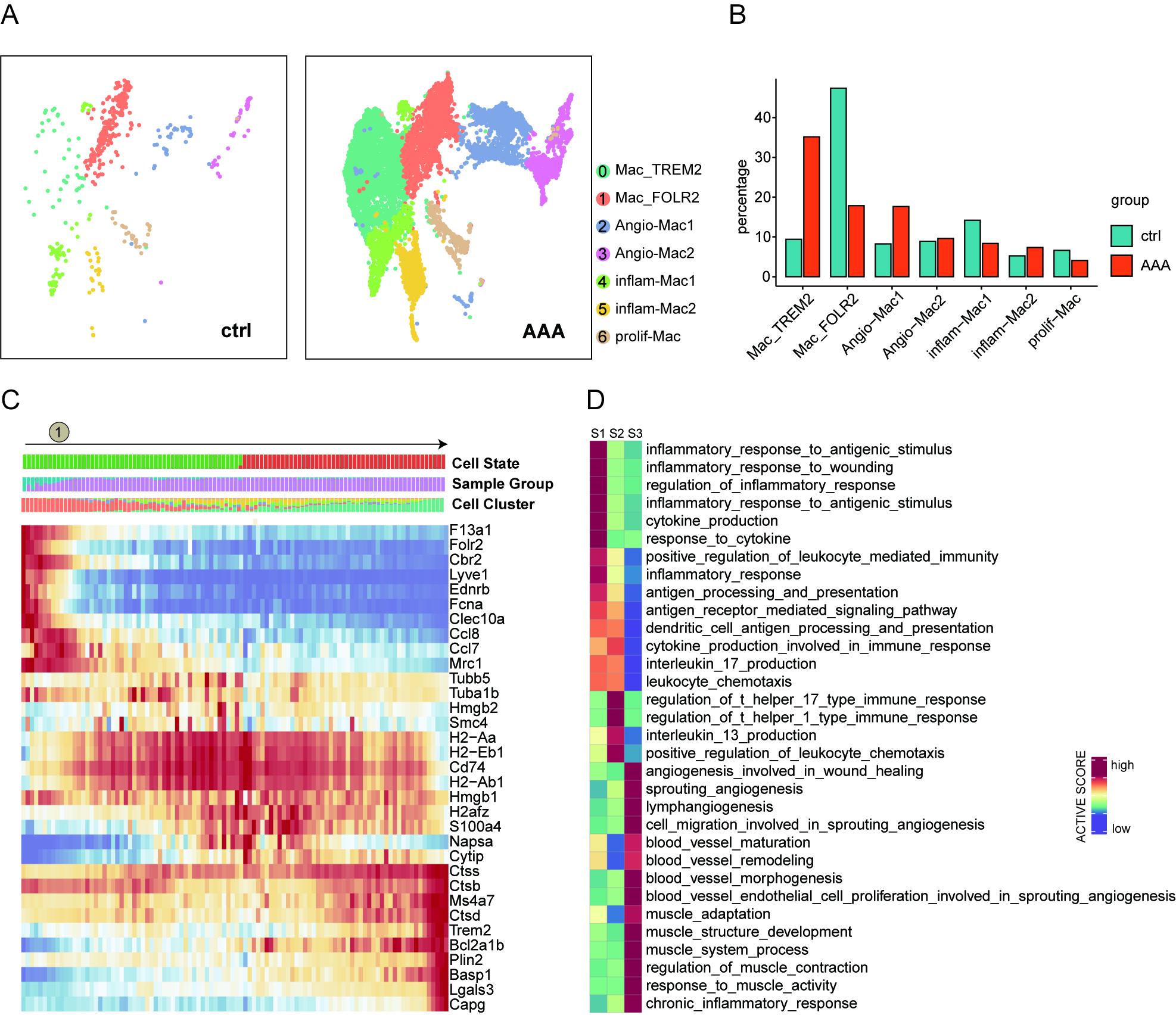

Supplement: Supplementary Figure 5 — (A) UMAP visualization of macrophages derived from normal and AAA’s abdominal aorta. (B) Bar plot showing subtypes of macrophage abundance for samples from different groups. (C) Heatmap illustrating the gene dynamics of macrophages along the pseudotime of route 2. Bar plots above the heatmap are scaled diagrams of different cell states, sample groups, and cell clusters during pseudotime differentiation trajectory. (D) Heatmap showing the functional pathways enriched in three cell states (S1–S3) of macrophages by GSVA analysis. [file Image5.tif]

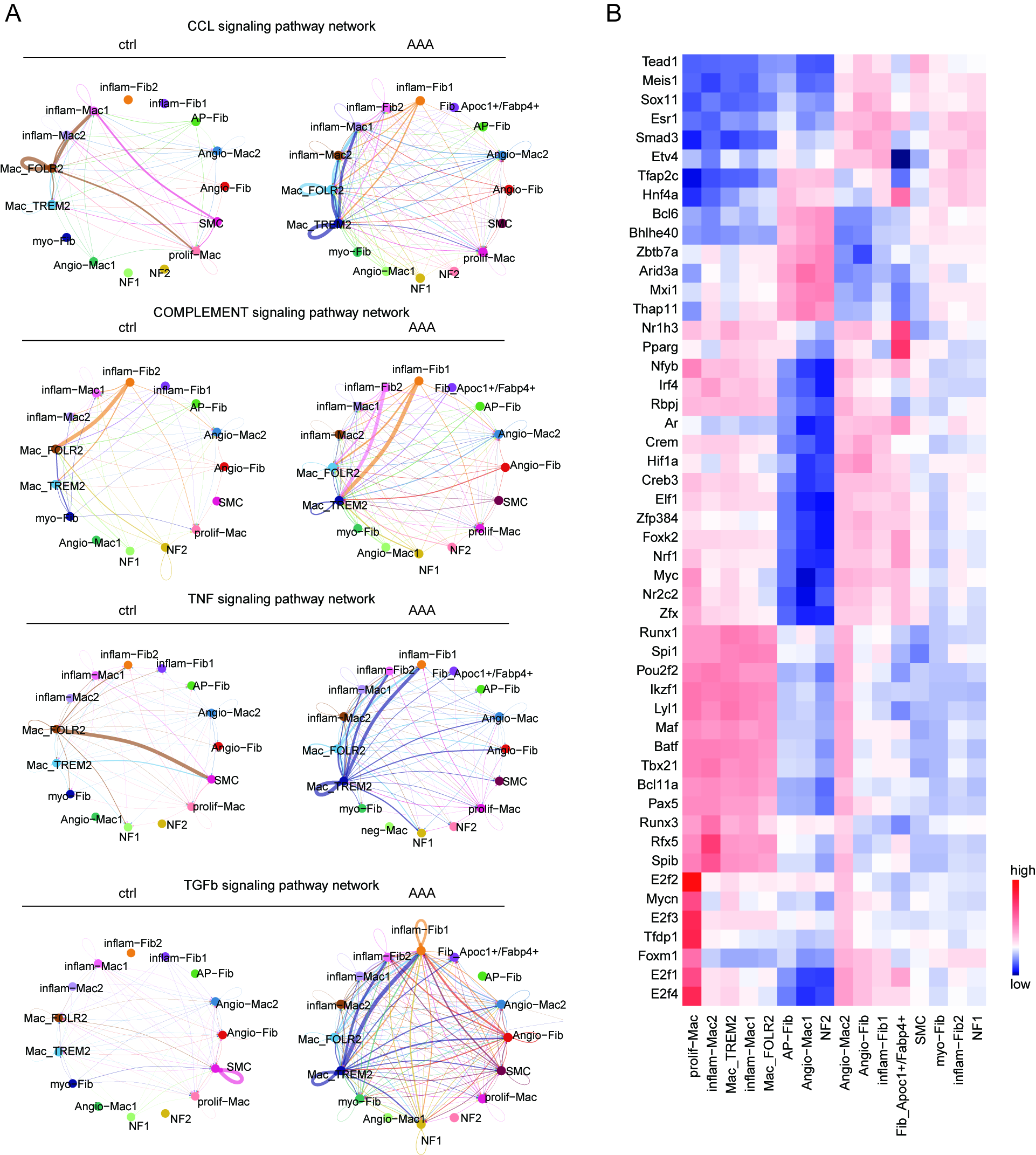

Supplement: Supplementary Figure 6 — (A)Circle plots showing the enhancement of CCL-/COMPLEMENT-/TGFb-/TNF- signaling network for each of subtypes in control (ctrl) and AAA’s (AAA) abdominal aorta. Each colored dot represents individual cell types, and the width of edges connecting circles represents the strength of intercellular signaling. (B) Heatmap showing the top 50 hub genes regulating specific genetic programs during AAA formation. [file Image6.tif]
